# Clinical Effectiveness of N-Methyl-D-Aspartate (NMDA) Receptor Antagonists in Adult Obsessive-Compulsive Disorder (OCD) Treatment: A Systematic Review

**DOI:** 10.7759/cureus.37833

**Published:** 2023-04-19

**Authors:** Asila A Ferguson, Aujala Irfan Khan, Baraa Abuzainah, Dipabali Chaudhuri, Kokab Irfan Khan, Roba Al Shouli, Akhil Allakky, Jaafar A Hamdan

**Affiliations:** 1 Psychiatry, California Institute of Behavioral Neurosciences & Psychology, Fairfield, USA; 2 Research, California Institute of Behavioral Neurosciences & Psychology, Fairfield, USA; 3 General Practice, California Institute of Behavioral Neurosciences & Psychology, Fairfield, USA; 4 Pediatric, California Institute of Behavioral Neurosciences & Psychology, Fairfield, USA; 5 Internal Medicine, California Institute of Behavioral Neurosciences & Psychology, Fairfield, USA; 6 Medicine, American University of Antigua, St. John, ATG

**Keywords:** amantadine, esketamine, ketamine infusion, memantine, nmda receptor antagonist, obsessive-compulsive disorders

## Abstract

Obsessive-compulsive disorder (OCD) is a neuropsychiatric disorder that affects approximately 2% of the human population. Traditional treatment of OCD includes selective serotonin reuptake inhibitor (SSRI) or serotonin reuptake inhibitor (SRI) treatment along with cognitive behavioral therapy (CBT). Nearly 25%-30% of OCD patients do not respond to SSRIs. Glutamatergic agents are currently being studied for the treatment of OCD due to the glutamatergic pathway in the brain, related to OCD, and the role of the cortico-striato-thalamic circuit (CSTC). This review assesses the clinical effectiveness of N-methyl-D-aspartate (NMDA) antagonists, ketamine/esketamine, memantine, and amantadine, for adult patients with OCD. Inclusion criteria include human studies published within the last 15 years, with patients diagnosed with OCD, aged over 18 years, with only psychiatric comorbidities, and full-text articles. Papers that included interventions other than CBT, exposure with response prevention (ERP), and SSRI/SRI were excluded. Articles were searched for using PubMed, PubMed Central, Medical Literature Analysis and Retrieval System Online, GeorgiA LIbrary LEarning Online, EBSCO Information Services, OpenAthens, Multidisciplinary Digital Publishing Institute, and Google Scholar databases, last searched on December 2, 2022. The risk of bias was assessed using Cochrane Risk of Bias tools, the Scale for the Assessment of Narrative Review Articles (SANRA) checklist for literature reviews, and the Joanna Briggs Institute (JBI) Critical Appraisal Checklist for quasi-experimental studies. Results were presented and synthesized by Excel spreadsheet analysis. The database search yielded 4,215 articles, which was cut down to 18 articles by inclusion/exclusion criteria, including duplications. 80% of the ketamine studies resulted in a significant reduction of obsessions and compulsions based on the Yale-Brown Obsessive-Compulsive Scale (Y-BOCS), and each of the memantine and amantadine studies displayed clinical effectiveness, also. Limitations include the small number of amantadine studies and the limited availability of other NMDA receptor (NMDAR) antagonist-focused studies. This systematic review shows that ketamine is an effective drug for the treatment of non-refractory, mild to moderate OCD, and memantine and amantadine are effective augmentation agents for the treatment of mild to severe OCD.

## Introduction and background

Obsessive-compulsive disorder (OCD) is a psychiatric disorder recognized by the fifth edition of the Diagnostic and Statistical Manual of Mental Disorders (DSM-5) that is characterized by having uncontrollable obsessions and/or compulsions. OCD used to be classified as an anxiety disorder (up until the fifth edition of the diagnostic manual) due to the increased awareness of OCD-type disorders and their unique identifiers that separate them from anxiety disorders. Many patients with OCD are aware that their symptoms of repetitive behaviors and intrusive thoughts are hyperreactive presentations of reality. This awareness usually aggravates the patient, as treating and managing symptoms alone is often unhelpful [1,2]. Finding effective methods to alleviate symptoms for patients with OCD is a global health priority, as approximately 2% of the global population is affected [3]. OCD is one of the top 10 disorders that cause disability, according to the World Health Organization [4]. Through the investigation of the pathological mechanism of OCD, it has been found that the glutamatergic system in the brain might play a significant role [5].

Glutamate is an important excitatory neurotransmitter that is involved in cell signaling in the nervous system. Increased glutamate concentration in cerebrospinal fluid (CSF) in brain areas, such as the prefrontal cortex, is associated with OCD [5,6]. Glutamate receptors are essential for neurotransmitters to stimulate signal transmission between axon terminals. N-methyl-D-aspartate (NMDA) receptors (NMDAR) are ionotropic glutamate receptors that create the framework for understanding the mechanism of action of the chemical agents focused on in this review: ketamine, esketamine, memantine, and amantadine. Ionotropic glutamate receptors are associated with the short-term/immediate effects of drugs that augment these receptors. For example, ketamine is known for its immediate and short-lasting improvement of OCD symptoms following intravenous infusion [7,8]. The other glutamatergic agents used in the neurologic, psychologic, and analgesic sects of medicine include riluzole, glycine, topiramate, lamotrigine, N-acetyl cysteine, and D-cycloserine, which either partially or completely affect the glutamatergic system and the binding site of glutamate receptors [5].

Cortico-striato-thalamo-cortical (CSTC) circuit dysfunction in the central nervous system has been attributed to OCD in several studies [5,9-11]. According to Ting and Feng, the CSTC begins as cortical neuron dendrites reach the striatum and synapse onto medium-spiny neurons. Then, γ-aminobutyric acid-(GABA)-ergic neurons connect to the globus pallidus pars internalis and substantia nigra pars reticulata. Two dopamine receptor types, types 1 and 2, comprise the direct and indirect pathways of expression, respectively. In the direct, or striatonigral pathway, the axons terminate in the globus pallidus pars internalis and substantia nigra, and in the indirect pathway, axons involve the globus pallidus pars externalis and the subthalamic nucleus, then return to their origin via the circuit loop [12,13]. Glutamate transmission abnormalities in the CSTC show evidence of playing a role in the pathophysiology of OCD [5]. Reduction in signal transmission from inhibitory nuclei in the direct pathway causes increased thalamic output to the cortex [14]. The indirect pathway modulates glutamatergic transmission by inhibiting the thalamus [15]. Glutamatergic agents are also glutamate modulators, and thus affect signal transmission. Genes coding for NMDAR subunits have been linked to OCD [5]. So, NMDAR antagonists, which target NMDAR, have the potential to modulate the activity of the glutamatergic pathways of the CSTC, which is why pharmacological trials should continue for the treatment of OCD.

Conventional treatments of OCD include psychological treatments, such as exposure with response prevention (ERP) therapy and cognitive behavioral therapy (CBT), which are recommended by the National Institute for Health and Care Excellence (NICE) CG31 guidelines [16,17]. They also include treatment with selective serotonin reuptake inhibitors (SSRIs) and serotonin reuptake inhibitors (SRIs). Stepped care approaches, which are combination treatments, are recommended for patients who do not respond to SSRI alone or CBT (including ERP) alone, and for patients with severe functional impairment [17]. The American Psychiatric Association lists first-line treatments of OCD as CBT, SSRI, and CBT + SSRI [18]. Low-intensity psychological treatments are recommended for patients with mild functional impairment, and patients with severe functional impairment are recommended to combine CBT/ERP with SSRI treatment [16,17]. The limitations of these first-line treatments mainly are that 25%-30% of patients diagnosed with OCD are refractory to SSRI treatment [5]. Limitations of SSRI/SRI treatments are also attributed to inaccurate diagnosis, inappropriate medication (comorbidity with bipolar disorder indicates against SRI treatment), or insufficient pharmacological study [19]. SRI-resistant patients are often recommended a trial of clomipramine or an antipsychotic agent (ex. quetiapine) [16,19]. Dopamine antagonists are being used as an alternative treatment for some patients who are SSRI/SRI-refractory, but there are significant risks and low rates of efficacy that deter the use of dopamine antagonists [19]. So, glutamatergic agents are currently being researched, as they have lower risk profiles and show evidence of anti-obsessive-compulsory action [19]. Due to their pharmacological benefit, glutamatergic agents such as NMDAR antagonists should be studied for their benefit in treating OCD.

Ketamine is an extrasynaptic NMDAR open-channel blocker, for which the mechanism of action is not significantly influenced by glutamate concentration [20]. Ketamine stimulates recovery of GluN1/2B receptor desensitization and inhibits the receptors more efficiently when the exposure rate to glutamate is low [20]. This mechanism perhaps explains why the psychological effects of ketamine are usually short-term, which has been demonstrated in many clinical trials and case studies [8]. Ketamine has been hypothesized to reduce OCD-like behavior, in a short-lasting manner, in low doses [8]. However, depressive symptoms are usually those that respond faster to ketamine than OCD symptoms. This is hypothesized to be due to ketamine’s activation of the α-amino-3-hydroxyl-5-methyl-4-isoxazole propionic acid receptor (AMPAR) and inhibition of the NMDAR [21,22]. Ketamine and esketamine administration have also been found to alter dopamine levels in the brain, which also may be related to its anti-depressive and anti-obsessive/compulsive effects [23].

Memantine is a synaptic NMDAR open-channel blocker that stabilizes a calcium-dependent desensitized state of GluN1/2A receptors upon binding [20]. The action of inhibition has a proportionate relationship with the frequency of glutamate exposure, but no obvious relationship with glutamate concentration at the synaptic cleft [24]. Memantine has been used as an augmenting agent for OCD treatment alongside CBT, particularly with patients who are refractory to traditional treatment [5,25-27].

Amantadine is a noncompetitive NMDAR antagonist that expresses the potential to reduce OCD symptoms by adjunction with SSRIs [9]. It is also a dopaminergic agonist commonly used as an antiviral agent [28,29].

There are several ongoing clinical trials that connect the glutamatergic system with the pathogenesis and pathophysiology of OCD. There are still discoveries to be made regarding the glutamatergic pathway’s role in OCD, which is why neurologists and psychiatrists continue to perform research to isolate this role. N-methyl-D-aspartate receptor antagonists show promise to treat moderate to severe OCD, along with other glutamatergic agents. Other glutamatergic agents were not included in the review due to the scale of the study and limited available data. In this paper, we will analyze the medical efficacy of NMDAR antagonists, ketamine, memantine, and amantadine, for the treatment of OCD through available studies, using the Yale-Brown Obsessive-Compulsive Scale (Y-BOCS), which is used to measure the severity of OCD. 

Methods

This systematic review was conducted using the Preferred Reporting Items for Systematic Reviews and Meta-Analyses (PRISMA) guidelines [30].

Inclusion/Exclusion Criteria

The literature search was performed to isolate papers that illuminate the clinical effectiveness of using the NMDAR antagonists memantine, amantadine, ketamine, and/or esketamine to treat OCD in adult patients. To achieve this, inclusion criteria were restricted to human studies published within the last 15 years focused on an adult population (18 years +), patients diagnosed with OCD and allowed only psychiatric comorbidities, and full-text papers limited to clinical trials, case studies, case series, meta-analyses, systematic reviews, literature reviews, case controls, cross-sectional studies, cohort studies, and in vitro crossover trials. Papers including interventions other than CBT/ERP and SRIs/SSRIs used in conjunction with an NMDA antagonist were excluded.

Data Source and Strategy

In the first search dated August 21, 2021, the PubMed search was performed according to Table 1. It yielded 4,215 results initially. After the year’s exclusion criteria, there were 2,257 studies. Followed by the “free full text” criterion, another 1,523 articles were excluded. The humans-only study criterion resulted in 516 studies. The age exclusion yielded 273 studies. Eleven studies were excluded based on language. Then, a title screen yielded five results. 11,800 results were yielded through the Google Scholar search. The year exclusion resulted in 9,840 results. The title screen originally resulted in 12 articles, and four duplicates were removed to equate to eight results. The EBSCO Information Services/GeorgiA LIbrary LEarning Online (EBSCO/GALILEO) search resulted in 119,605 results. After articles other than full texts were excluded, the result list included 93,504 articles. The language criterion yielded 90,381 results. Academic journal source type restricted it to 74,182 articles. Then, the date restriction yielded 58,548 results. A manual search and title screen were performed due to there not being enough filters to adhere to the chosen inclusion and exclusion criteria. Many papers were not related to the topic, involved Parkinsonism disease, and/or were not focused on human subjects. This search and screen yielded four results. All searches were performed independently, by one reviewer. The MDPI search resulted in one article, making a total of 18 studies. A second search was performed on December 2, 2022, following the same PubMed search strategy as indicated in Table 1. The PubMed search was performed according to Table 1. The initial result total was 4,390. The year criterion included 2,278 articles. Followed by the “free full text” criterion, the search yielded 787 results. Then, restricted to humans, it included 540 results. The age exclusion resulted in 269 articles. Language excluded 10 more articles. After article-type screening, 132 results were left. The title screen yielded six results. Out of these six, one of the articles was new to this review. It was excluded because it was a study focused on the method of administration of ketamine, instead of the overall clinical effectiveness of ketamine as a treatment for OCD. The EBSCO/GALILEO search was not performed again as the author no longer had access to the database. The MDPI search initially resulted in 199 results. The year did not change the total. Exclusion by article type resulted in 66 articles. After the title screen, there were no results. The search was performed again several times with different keyword combinations, with the same results. All searches were performed independently, by one reviewer.

**Table 1:** PubMed search strategy Concepts were searched for by combining keywords with the Boolean term “OR”. Restrictions to Medical Subject Headings (MeSH) major topic were applied, then concepts were combined with the Boolean term “AND” for the final algorithm. NMDA stands for N-methyl-D-aspartate and NMDA receptor antagonist MN-08 is a synthetic NMDAR antagonist.

Concept	Keywords	PubMed Search Builder	Full MeSH Algorithm	Yield
Obsessive compulsive disorder	Obsessive-compulsive disorder, compulsions, obsessive behavior, anxiety disorder	((((( "Obsessive-Compulsive Disorder/drug therapy"[Majr] OR "Obsessive-Compulsive Disorder/prevention and control"[Majr] OR "Obsessive-Compulsive Disorder/psychology"[Majr] )) OR ( "Obsessive Behavior/drug therapy"[Majr] OR "Obsessive Behavior/prevention and control"[Majr] OR "Obsessive Behavior/psychology"[Majr] )) OR ( "Compulsive Behavior/drug therapy"[Majr] OR "Compulsive Behavior/prevention and control"[Majr] OR "Compulsive Behavior/psychology"[Majr] )) OR ( "Obsessive-Compulsive Disorder/rehabilitation"[Majr] OR "Obsessive-Compulsive Disorder/therapy"[Majr] )) OR ( "Anxiety Disorders/drug therapy"[Mesh] OR "Anxiety Disorders/prevention and control"[Mesh] OR "Anxiety Disorders/psychology"[Mesh] )	((((( "Obsessive-Compulsive Disorder/drug therapy"[Majr] OR "Obsessive-Compulsive Disorder/prevention and control"[Majr] OR "Obsessive-Compulsive Disorder/psychology"[Majr] )) OR ( "Obsessive Behavior/drug therapy"[Majr] OR "Obsessive Behavior/prevention and control"[Majr] OR "Obsessive Behavior/psychology"[Majr] )) OR ( "Compulsive Behavior/drug therapy"[Majr] OR "Compulsive Behavior/prevention and control"[Majr] OR "Compulsive Behavior/psychology"[Majr] )) AND ( "Obsessive-Compulsive Disorder/rehabilitation"[Majr] OR "Obsessive-Compulsive Disorder/therapy"[Majr] )) OR ( "Anxiety Disorders/drug therapy"[Mesh] OR "Anxiety Disorders/prevention and control"[Mesh] OR "Anxiety Disorders/psychology"[Mesh] ) AND (( "Ketamine/antagonists and inhibitors"[Majr] OR "Ketamine/pharmacokinetics"[Majr] OR "Ketamine/pharmacology"[Majr] OR "Ketamine/therapeutic use"[Majr] )) OR "Esketamine" [Majr] OR ( "Memantine/antagonists and inhibitors"[Mesh] OR "Memantine/pharmacokinetics"[Mesh] OR "Memantine/pharmacology"[Mesh] OR "Memantine/therapeutic use"[Mesh] OR "N-methyl-d-aspartate receptor antagonist MN-08" [Majr]) OR ( "Amantadine/antagonists and inhibitors"[Majr] OR "Amantadine/pharmacokinetics"[Majr] OR "Amantadine/pharmacology"[Majr] OR "Amantadine/therapeutic use"[Majr] )	This search yielded 4,221 articles.
NMDA receptor antagonists	Ketamine, esketamine, memantine, n-methyl-d-aspartate receptor antagonist MN-08, amantadine	(( "Ketamine/antagonists and inhibitors"[Majr] OR "Ketamine/pharmacokinetics"[Majr] OR "Ketamine/pharmacology"[Majr] OR "Ketamine/therapeutic use"[Majr] )) OR "Esketamine" [Majr] OR ( "Memantine/antagonists and inhibitors"[Mesh] OR "Memantine/pharmacokinetics"[Mesh] OR "Memantine/pharmacology"[Mesh] OR "Memantine/therapeutic use"[Mesh] OR "N-methyl-d-aspartate receptor antagonist MN-08" [Majr]) OR ( "Amantadine/antagonists and inhibitors"[Majr] OR "Amantadine/pharmacokinetics"[Majr] OR "Amantadine/pharmacology"[Majr] OR "Amantadine/therapeutic use"[Majr] )		

**Figure 1:**
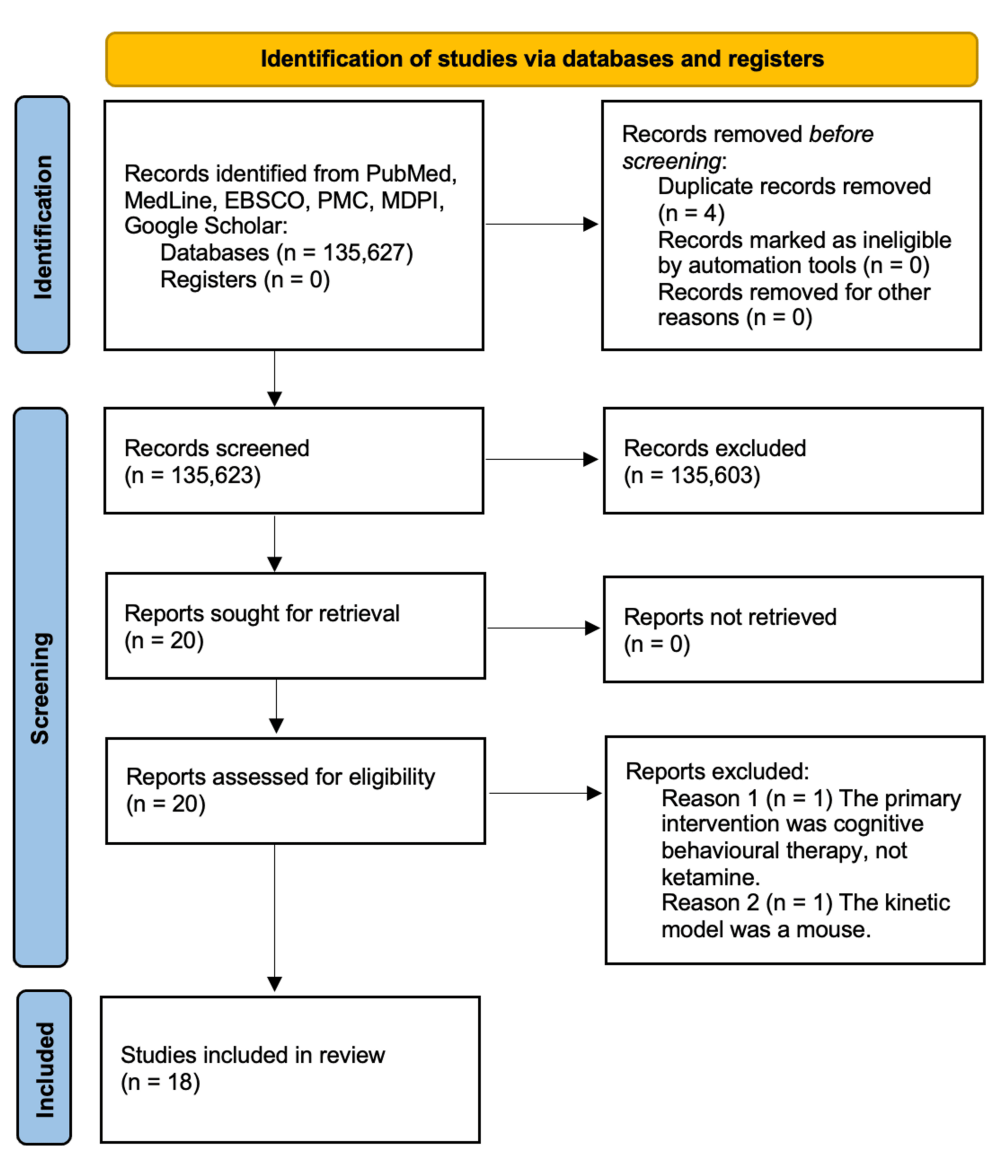
PRISMA flow diagram The Preferred Reporting Items for Systematic Reviews and Meta-Analyses (PRISMA) flow diagram shows the searching process for this systematic review. The databases include PubMed, PubMed Central (PMC), Medical Literature Analysis and Retrieval System Online (MedLine), GeorgiA LIbrary LEarning Online (GALILEO), EBSCO Information Services (EBSCO), OpenAthens, Multidisciplinary Digital Publishing Institute (MDPI) and Google Scholar.

Table 1 shows how data were extracted from research databases. Research papers were extracted from PubMed, PubMed Central (PMC), Medical Literature Analysis and Retrieval System Online (MedLine), GALILEO, EBSCO, OpenAthens, Multidisciplinary Digital Publishing Institute (MDPI) and Google Scholar. Research in these databases was performed on August 21, 2021. The concepts used to locate eligible articles via PubMed search included (“Obsessive compulsive disorder” and “NMDA receptor antagonists”). The search was complemented with keywords via the Boolean term “OR” after the use of Medical Subject Headings (MeSH) such as “antagonists and inhibitors,” “therapeutic use,” “pharmacokinetics,” and “pharmacology.” Figure 1 indicates how data for the systematic review were retrieved under the PRISMA 2020 guidelines.

Bias Evaluation Tools

Quality analyses were performed to demonstrate the risk of bias in the selected articles using checklists and software, including Joanna Briggs Institute (JBI) critical appraisals, Cochrane risk of bias tools, and the Scale for the Assessment of Narrative Review Articles (SANRA), as demonstrated in Table 2.

**Table 2:** Quality appraisal Papers with less than 30% risk of bias were included in this systematic review. The bias evaluation tools include the Joanna Briggs Institute (JBI) quality appraisals, Cochrane risk of bias (RoB), and the Scale for the Assessment of Narrative Review Articles (SANRA).

Article	Bias Evaluation Tool	Non-Bias Percentage	Included/Excluded
Murphy DL, Timpano KR, Wheaton MG, Greenberg BD, Miguel EC. Obsessive-compulsive disorder and its related disorders: a reappraisal of obsessive-compulsive spectrum concepts. Dialogues in Clinical Neuroscience. 2010;12(2):131-48. [1]	SANRA- A Scale for the Quality Assessment of Narrative Review Articles [31]	100%	Included
Sheshachala K, Narayanaswamy J. Glutamatergic augmentation strategies in obsessive-compulsive disorder. Indian Journal of Psychiatry. 2019;61:S58-S65. doi: 10.4103/psychiatry.IndianJPsychiatry_520_18. [5]	SANRA- A Scale for the Quality Assessment of Narrative Review Articles [31]	83.3%	Included
Rodriguez CI, Kegeles LS, Flood P, Simpson HB. Rapid Resolution of Obsessions After an Infusion of Intravenous Ketamine in a Patient with Treatment-Resistant Obsessive-Compulsive Disorder. Journal of Clinical Psychiatry. 2011;72(4). [7]	Joanna Briggs Institute 2017 Critical Appraisal Checklist for Case Reports [32]	100%	Included
Naderi, S.F., H.; Aqamolaei, A.; Mortazavi, S. H.; and A.S. Mortezaei, E.; Rezaei, F., Akhondzadeh, S., Amantadine as adjuvant therapy in the treatment of moderate to severe obsessive–compulsive disorder- A double-blind randomized trial with placebo control. Psychiatry and Clinical Neurosciences, 2019. 73: p. 169–174. [9]	Cochrane risk of bias tool for randomized trials [33]	100%	Included
Martinotti G, Chiapinni S, Pettorruso m, Mosca A, Miuli A, Di Carlo F, et al. Therapeutic Potentials of Ketamine and Esketamine in Obsessive-Compulsive Disorder (OCD), Substance Use Disorders (SUD) and Eating Disorders (ED): A Review of the Current Literature. Brain Sciences. 2021;11(856). doi: 10.3390/brainsci11070856. [23]	SANRA- A Scale for the Quality Assessment of Narrative Review Articles [31]	91.7%	Included
Aboujaoude E, Burry JJ, Gamel N. Memantine Augmentation in Treatment-Resistant Obsessive-Compulsive Disorder. Journal of Clinical Psychopharmacology. 2009;29(1):51-5. doi: 10.1097/JCP.0b013e318192e9a4. [25]	Joanna Briggs Institute 2017 Critical Appraisal Checklist for Quasi-Experimental Studies [32]	88.9%	Included
Modarresi A, Chaibakhsh S, Koulaeinejad N, Koupaei SR. A systematic review and meta-analysis: Memantine augmentation in moderate to severe obsessive-compulsive disorder. Psychiatry Research. 2019;282. doi: 10.1016/j.psychres.2019.112602. [26]	SANRA- A Scale for the Quality Assessment of Narrative Review Articles [31]	100%	Included
Pasquini M, Biondi M. Memantine augmentation for refractory obsessive-compulsive disorder. Progress in Neuro-Psychopharmacology & Biological Psychiatry. 2006;30:1173-5. doi: 10.1016/j.pnpbp.2006.04.013. [27]	JBI 2017 Critical Appraisal Checklist for Case Series [32]	100%	Included
Stryjer R, Budnik D, Ebert T, Green T, Polak L, Weizman S, et al. Amantadine Augmentation Therapy for Obsessive Compulsive Patients Resistant to SSRIs - An Open-Label Study. Clinical Neuropharmacology. 2014;37:79-81. doi: 10.1097/WNF.0000000000000029. [29]	Joanna Briggs Institute 2017 Critical Appraisal Checklist for Quasi-Experimental Studies [32]	88.9%	Included
Stewart, S.E., et al., A single-blinded case-control study of memantine in severe obsessive-compulsive disorder. J Clin Psychopharmacol, 2010. 30(1): p. 34-9. [34]	Cochrane risk of bias tool for randomized trials [33]	88.3%	Included
Ghaleiha, A., et al., Memantine add-on in moderate to severe obsessive-compulsive disorder: randomized double-blind placebo-controlled study. J Psychiatr Res, 2013. 47(2): p. 175-80. [35]	Cochrane risk of bias tool for randomized trials [33]	100%	Included
Bloch M, Wasylink S, Landeros-Weisenberger A, Panza KE, Billingslea E, Leckman JF, et al. Effects of Ketamine in Treatment-Refractory Obsessive-Compulsive Disorder. Biological Psychiatry. 2012;72(11):964-70. doi: 10.1016/j.biopsych.2012.05.028. [36]	JBI Critical Appraisal Checklist for Quasi-Experimental Studies [32]	85.7%	Included
Rodriguez CI, Kegeles LS, Levinson A, Feng T, Marcus SM, Vermes D, et al. Randomized Controlled Crossover Trial of Ketamine in Obsessive-Compulsive Disorder: Proof-of-Concept. Neuropsychopharmacology. 2013;38:2475-83. doi: 10.1038/npp.2013.150. [37]	Cochrane Risk of Bias 2 for crossover trials [33]	90%	Included
Adams, T.G., M.H. Bloch, and C. Pittenger, Intranasal Ketamine and Cognitive-Behavioral Therapy for Treatment-Refractory Obsessive-Compulsive Disorder. J Clin Psychopharmacol, 2017. 37(2): p. 269-271. [38]	JBI Critical Appraisal Checklist for Quasi-Experimental Studies [32]	83.3%	Included
Rodriguez CI, Levinson A, Zwerling J, Vermes D, Simpson HB. Open-Label trial on the effects of memantine in adults with obsessive-compulsive disorder after a single ketamine infusion. J Clin Psychiatry. 2016;77(5):688-9. doi: 10.4088/JCP.15l10318. PubMed PMID: 27249077; PubMed Central PMCID: PMC5544938. [39]	Joanna Briggs Institute 2017 Critical Appraisal Checklist for Quasi-Experimental Studies [32]	88.9%	Included
Grassi G, Cecchelli C, Vignozzi L, Pacini S. Investigational and Experimental Drugs to Treat Obsessive-Compulsive Disorder. Journal of Experimental Pharmacology. 2020;12:695-706. doi: 10.2147/JEP.S255375. [40]	SANRA- A Scale for the Quality Assessment of Narrative Review Articles [31]	100%	Included
Rodriguez, C.I., et al., In vivo effects of ketamine on glutamate-glutamine and gamma-aminobutyric acid in obsessive-compulsive disorder: Proof of concept. Psychiatry Res, 2015. 233(2): p. 141-7. [41]	Cochrane risk of bias tool for randomized trials [33]	90%	Included
Modarresi A, Sayyah M, Razooghi S, Eslami K, Javadi M, Kouti L. Memantine Augmentation Improves Symptoms in Serotonin Reuptake Inhibitor-Refractory Obsessive-Compulsive Disorder: A Randomized Controlled Trial. Pharmacopsychiatry. 2018;51:263-9. doi: 10.1055/s-0043-120268. [42]	Cochrane risk of bias tool for randomized trials [33]	100%	Included

Results

Eighteen studies consisting of randomized controlled trials (RCTs), open-label studies, cohort studies, case-control studies, systematic reviews, meta-analyses, and literature reviews were used to collect data for this systematic review. These studies focused on the clinical effectiveness of NMDAR antagonists, particularly ketamine/esketamine, amantadine, and memantine, in the treatment of OCD. Clinical improvement from intervention was usually standardized to at least a 35% reduction in Y-BOCS score, though, in one study, the metric was at least 25% [34]. Excluding the literature reviews used to conduct this review, there were 297 patients in 14 studies who were identified with OCD. The results demonstrated significant decreases in Y-BOCS (13 studies) and OCD Visual Analog Scale (OCD-VAS) scores (one study) in the 14 studies. In 12 of these studies, the clinical effectiveness of the NMDAR antagonist was reached, though there were varying results within the studies. The literature reviews indicated that glutamatergic agents such as amantadine, ketamine/esketamine, memantine, riluzole, glycine, topiramate, lomatrigine, N-acetyl cysteine, and D-cycloserine have been studied or are planned to be studied in the context of OCD research [5,41].

Ketamine

Six out of 14 of the primary research studies studied ketamine as a treatment for mostly refractory OCD. This includes the study that used memantine and ketamine as OCD treatments/interventions [39]. This study's intervention was a single intravenous (IV) dose of ketamine, PO 5 mg memantine daily titrated by 5 mg weekly to 10 mg twice daily for up to six weeks, and memantine for a total of 12 weeks [39]. The results are as follows: eight of 12 participants had no ketamine response, and none of the participants showed significant Y-BOCS changes six weeks post memantine initiation (t_11=1.28, p = 0.23) [39]. 80% of the ketamine studies resulted in a significant decrease in Y-BOCS, and in one case, OCD-VAS, which is another diagnostic scale used to determine OCD severity. One study which used ketamine in treatment-refractory OCD only saw an acute response that lasted one to three days, so it did not qualify for our clinical effectiveness measure [38]. This open-label study treated OCD with a 0.5 mg/kg ketamine infusion over 40 mins, which resulted in significant acute and transient improvement in OCD severity, Y-BOCS improvement peaked at 11% and was statistically significant over days 1-3 post-infusion (Wilcoxon Rank Sum Test: T=-2.81, N=10, p=0.005), but no subject exhibited an *a priori* OCD response to ketamine infusion (pre-defined as >= 35% reduction in Y-BOCS total score) [38]. There was one literature review that studied the potential of ketamine and esketamine in OCD, substance use disorder (SUD), and eating disorder (ED) [23]. The interventions include intranasal (IN) 10 mg ketamine via atomizer (five 10 mg doses, 5 mg/nostril, over a 20-minute period) and two IV infusions given one week apart of saline or 0.5 mg/kg ketamine HCl; repeated 0.5 mg/kg ketamine infusion (2-10 times) over 40 min; 40-min single IV infusion of 0.5 mg/kg ketamine; 0.5 mg/kg ketamine IV infusion; IV infusion 0.5 mg/kg ketamine, IV infusion 0.5 mg/kg ketamine; 40-min IV infusion 0.5mg/kg ketamine; IN ketamine 50 mg [23]. The results reviewed include, in the same order as the interventions: three-point Y-BOCS reduction; minimal reduction in obsessions after the first infusion, complete cessation of obsessions during the second ketamine infusion; statistically significant reduction in OCD severity following ketamine infusion, 21% showed clinical response; OCD symptom statistically significant improvement in the first three days post-infusion with < 12% OCD response, no significant reduction in Y-BOCS; ketamine group 50% treatment response (>= 35% Y-BOCS reduction vs. 0% placebo group); no change in Glx, a significant increase in GABA/W post-infusion; no significant Y-BOCS-measured response to 12 weeks of memantine post-ketamine infusion; significant reduction in OCD severity over two weeks ERP; and no significant treatment response [23]. An RCT crossover trial with 15 participants gave two 40-minute IV infusions at least one week apart of either saline or 0.5 mg/kg ketamine resulted in the ketamine group having a lower mean estimated baseline OCD-VAS score at mid-infusion (-4.52 points, SE=1.23, p<0.005), 230 min (3.84 points, SE=1.59, p<0.05), and seven days post-infusion (-3.67 points, SE=1.36, p<0.05) than the placebo group, and a 50% treatment response in ketamine group versus a 0% response in the placebo group (chi-squared (1, N=15) = 4.77, p<0.05) [38]. One patient was treated with two IV infusions given one week apart of either saline or 0.5 mg/kg ketamine which resulted in a minimal reduction of obsessions during the first infusion (saline), and complete cessation of obsessions during the second infusion (ketamine) [7]. Her obsessions returned seven days later [7]. A study that assessed the effects of ketamine on OCD and on glutamate-glutamine (Glx-W) and gamma-aminobutyric acid (GABA) concentrations in the brain treated 16 patients with an IV infusion of 0.5 mg/kg ketamine, resulting in no significant difference between ketamine and saline conditions overtime in Glx/W (F=0.65, df=6,141, p=0.689); modest differences in GABA/W (F=2.16, df=6,146, p-0.048), similar results in the second phase with the first treatment phase: GABA/W significantly increased 60-73 minutes post-infusion ketamine vs. saline (t(146)=2.38, p=0.02) and ketamine vs. baseline (t(72)=2.22, p=0.03); change in GABA/W from baseline to previous time point (60 < t < 73) was positively correlated with changes in OCD-VAS score during and after infusion up to day 7 (R-values between 0.46 and 0.63, p-values between 0.01 and 0.06); and a significant decrease in OCD-VAS score [41]. Lastly, a case report treating treatment-refractory OCD with IN ketamine resulted in a Y-BOCS total score reduction from 28 to 20 after one week which varied after seven days post-treatment [38].

Memantine

Six out of 14 of the primary research studies studied memantine as either the sole intervention or as an adjuvant/augment therapy. In these studies, there was a significant decrease in Y-BOCS after memantine administration, though the proportion of patients who achieved partial or full remission varied based on the experiment [23,25,27,34,35,37,43]. A single-blinded case-control study of memantine in severe OCD treated 44 patients with a mean starting dose of 5 mg memantine increased to a final dose of 18 mg [34]. It resulted in a mean Y-BOCS decrease of 7.2 (6.4) points in the experimental group, or 27%, which meets >=25% decrease for clinical response, and 4.6 (5.9) for controls, or a 16.5% decrease; case group responders were significantly more likely to have a 50% OCD severity decrease than the control group (22.7% of case group vs. 4.5% of control group, chi-squared=4.27, p=0.04) [34]. Modaressi et al. performed an RCT for SRI-refractory OCD with a treatment of 20 mg/d memantine PO, resulting in a mean reduction in total Y-BOCS score of 40.9%; 73.3% of patients achieving a clinically significant response, and time x group ANOVA results: Time: F(2.1,57.9)=191.0, p < 0.001, n^2=0.87 (L) Group: F(1,28)=16.5, p < 0.001, n^2=0.37 (L) Time x Group: F(2.1,57.9)=189.8, p < 0.001, n^2 = 0.87 (L). A case report of two patients receiving memantine treatment for the treatment of refractory OCD, which did not specify the dose of memantine given, resulted in significant reductions in total Y-BOCS (28 +/- 4.5 vs. 18.8 +/- 8.8, p < 0.01, df=7, t=2.36) [36]. A double-blinded RCT that added memantine onto the current psychiatric medication regimen treated participants with 10 mg/d memantine in the first week then 20 mg/d for a total of eight weeks and 100 mg/d fluvoxamine for the first four weeks titrated afterward to 200 mg/d, or placebo + fluvoxamine (same concentration) [35]. It resulted in 89% of participants in the memantine group compared with 32% of participants in the placebo group achieving remission at the end of the trial (chi-squared(1)=13.328, p<0.001); repeated-measure ANOVA found a significant effect for time (Greenhouse-Geisser corrected: F(2.096, 75.470)=68.461, p<0.001) and for time x treatment interaction (Greenhouse-Geisser corrected: F(2.096, 75.470)=5.280, p=0.006) [35]. An SR/MA reviewed the treatments of 125 OCD patients with memantine [26]. Memantine PO was given -- this was inferred as there was no indication of dosage or other treatment details -- which resulted in a mean difference in Y-BOCS score change between memantine and placebo groups of 7.76 (95% CI: 2.58-12.95, p < 0.001); treatment response in memantine group was 81% and 19% in the placebo group [26]. These results were determined from eight different studies, and the Y-BOCS reduction was similar in SRI-refractory patients and non-refractory patients, with less reduction in comorbid patients [26]. The authors note a high risk of bias and a lack of diversity of patients due to the study location (Iran) [26]. An open-label study assessing the effect of memantine on treatment-refractory OCD treated 15 patients with 5 mg/d memantine increased in 5-mg increments weekly to a target dose of 10 mg BID for a total of 12 weeks, resulting in 42.9% of the treatment group meeting clinical response criteria (Y-BOCS reduction of at least 25%), and the mean Y-BOCS reduction was 45.9% (SD, 12.6%) [25]. Case reports of two patients treated with 5 mg/d memantine titrated for three weeks to 15 mg/d resulted in no significant Y-BOCS score reduction; the Y-BOCS score reduction was from 34 to 19 after three weeks of treatment [27].

Amantadine

Two primary research studies focused on amantadine as augmentation for currently medicated OCD patients. Both studies resulted in significant decreases in Y-BOCS, with 43 out of 50 patients in the RCT achieving remission [9,29]. The first study (open-label) treated SSRI-refractory subjects with 100 mg/d amantadine in the first week, which was increased to 200 mg/d in two divided doses for five weeks [29]. The second study (RCT) used amantadine as an adjuvant therapy and treated 100 patients with fluvoxamine (100 mg BID) and amantadine (100 mg/d) or fluvoxamine (100 mg BID) and placebo for 12 weeks [9]. It resulted in 22 of the amantadine group and 14 of the placebo group achieving remission and 43 of the amantadine group compared to 22 of the placebo group achieved complete or partial treatment response (p<0.001), repeated-measure ANOVA: significant effect for Time x Treatment interaction in Y-BOCS Obsession subscore (Greenhouse-Geisser corrected: F=3.84, df=1.50, p-0.03) [9].

## Review

Discussion

This review finds that NMDAR antagonists memantine, ketamine, and amantadine reduce OCD severity when used either solely or as an augment. Ketamine has the most varying results due to its short-lasting response to OCD symptoms [7,40]. It has been suggested to use ketamine in patients with non-refractory, mild to moderate OCD. Memantine and amantadine are effective augments and add-ons, and they lower the severity of OCD symptoms based on the Y-BOCS scale [5,9,25,26,29,42].

Review of OCD

OCD is a neuropsychiatric disorder characterized by intrusive obsessions and/or compulsions in affected patients [1,35]. Pathophysiological research demonstrates glutamatergic dysfunction in the CSTC that correlates with OCD and other related disorders such as anxiety disorder and EDs [1,5]. This prefaces the research in this article, which uses the knowledge of the glutamatergic pathway to select drugs (NMDAR antagonists) that attempt to specifically target OCD neurochemically, often after the failure of SSRI treatment. Ketamine stimulates the recovery of GluN1/2B receptor desensitization and inhibits receptors more efficiently when glutamate concentration is low [20]. As was mentioned previously, the research found that glutamate concentration is high in CSF of OCD patients, and in vivo research showed that glutamate concentration was dependent on ketamine, which may explain why ketamine’s effectiveness is short-lasting [5,41]. Memantine inhibits GluN1/2A receptors noncompetitively and stabilizes a calcium-dependent desensitized state of native NMDARs, which provides pharmaceutical neuroprotection [20]. Neuroprotection is also provided by the preferential inhibition of extrasynaptic NMDARs and the reduction of glutamatergic excitotoxicity [20,43,44]. Amantadine is a noncompetitive NMDAR antagonist that is similar to memantine, structurally and functionally [45].

Review of Studies

In an open-label trial testing the effects of ketamine for treatment-refractory OCD, there was no clinical improvement in Y-BOCS scores for the 10 patients studied [36]. Though, there was an acute response that lasted one to three days post-infusion [36]. A case report in which one patient with OCD received intranasal ketamine and CBT for six weeks resulted in a significant decrease in Y-BOCS [38]. Clinical improvement was achieved in most patients in an RCT -- over 50% of patients self-reported reduced obsessive and intrusive symptoms after intravenous (IV) ketamine infusion [37]. However, there was no statistical difference between OCD assessments comparing saline versus ketamine groups [38]. The in vivo trial that assessed both OCD symptoms and the relationship of GABA and glutamate with OCD and ketamine infusion found a significant decrease in OCD-VAS after ketamine infusion [41]. In this case, glutamate levels did not change after saline and ketamine infusions, though GABA had a positive relationship with ketamine [41]. This can provide further hypotheses about the appropriate approach to treating OCD, as it is known to play a role in the glutamatergic CSTC pathway. The last primary study was a case report of a patient who experienced rapid resolutions of obsessions after the second ketamine infusion, but obsessions returned seven days later [7].

Ketamine inhibits extrasynaptic ionotropic glutamate receptors, which contributes to an understanding of the acute and short-lasting response it causes in patients with OCD [20]. This does not explain, however, why other NMDAR antagonists last longer than ketamine. Ketamine's acute effect may be due to it affecting extrasynaptic glutamate receptors, while other drugs affect synaptic glutamate receptors [20]. A study found evidence of the GABA^B^ receptor gene on chromosome 6p21.3 region, which is an area that was previously linked to OCD [46]. This gives preliminary evidence that explains the increase in GABA levels after ketamine infusion in patients with OCD [41]. Ketamine also increases the activity of the glutamatergic pathway in the prefrontal cortex, which may explain its effect on the clinical representation of OCD [5,44]. Ketamine produces a greater effect in unmedicated, nonrefractory, less severe OCD patients based on the available study data [5]. These results show that ketamine can be used to lessen the severity of OCD in specific populations, with the knowledge that it produces an acute response in most patients.

As ketamine is known to be a potent, fast-acting anti-depressive and anti-suicidal agent [23,40]. There were varying inclusion and exclusion criteria in each of the studies, so some studies included patients with comorbidities, and others did not. Nonetheless, there was diversity within the patients’ comorbidities, and depression severity was not measured except for one study. Because of its effectiveness as an antidepressant, it is fair to explore whether ketamine can be used as an augmenting agent to SSRIs in patients with OCD and a comorbid depressive disorder, which could be tested with future research.

In a case-control study with 44 patients, half showed a statistically significant decrease in OCD severity after treatment with memantine [34]. In the open-label trial that assessed the effects of memantine after ketamine infusion, one out of 15 patients achieved a 34% reduction in Y-BOCS, and there were varying results among the study group [39]. An RCT of 32 OCD-diagnosed patients resulted in a 40.9% decrease in Y-BOCS, with a total of 73.3% of the patients achieving a clinical response to memantine [42]. A case report of two patients concluded with one patient receiving a clinical response to memantine [27]. Another RCT showed that 89% of the patients who received memantine had a significant decrease in Y-BOCS, compared to 32% in the placebo group [35]. An open-label experiment including 15 patients showed a mean 45.9% decrease in Y-BOCS [25]. The systematic review and meta-analysis chosen for this study included 125 patients, of whom there was a mean 39.5% reduction in Y-BOCS [26]. It reviewed eight studies in total which yielded that Y-BOCS reduction was similar in SRI-refractory and non-refractory patients, and there was an average lesser reduction in comorbid patients, in response to memantine intervention [26]. However, the authors note that the lack of diversity (all patients were Iranian) may have contributed to a biased review [26].

Memantine blocks synaptic NMDARs and is used as an augmenting agent alongside CBT for many patients with OCD [5,20,25,27,42]. It is suggested by neurologic research that memantine reduces the activity of the striatonigral (direct) pathway of the CSTC, which is a pathway implicated in the pathophysiology of OCD [15]. Memantine is a strong glutamatergic agent that continues to show great promise in reducing OCD severity for patients with severe OCD.

The first amantadine study used amantadine as an augment to SSRI-resistant patients with OCD, who were actively taking SSRIs, which resulted in a significant decrease in Y-BOCS [29]. However, it is unclear whether all patients (n = 8) had a similar reduction based on the available data [29]. The other study was similar and used amantadine as an adjuvant, which not only resulted in a significant decrease in Y-BOCS but also resulted in remission [9]. 86% of the amantadine group achieved remission, compared to 44% in the placebo group [9].

Amantadine is a weak noncompetitive NMDAR antagonist that has recently been used in obsessive-compulsive studies [29]. It has been used for the treatment of schizophrenia and Parkinson disease [43]. The data suggest that though it is a weaker NMDAR antagonist, it can serve as an effective augment to SSRI and CBT therapies. Future research should continue to test amantadine as a treatment for OCD.

Addition to the Medical Community

This review is one of the first to review three different glutamatergic agents’ effectiveness in treating OCD. It adds a cohesive review that analyzes the data and intervention outcomes for use by neurologists, psychiatrists, others in the medical field, and perhaps patients who are investigating different ways to treat their OCD. As 25%-30% of people with OCD do not respond to CBT and/or SSRI, which are traditional interventions for OCD, the results of this review provide possible effective agents for people with refractory OCD [5].

Limitations

We had several limitations in this review. The first limitation is that not all NMDAR antagonists were targeted in this review due to the large array of antagonists. NMDAR antagonists are part of a larger group known as glutamatergic agents, many of which have not yet been studied in abundance in this focus area. So, three NMDAR antagonists with the greatest number of studies, ketamine, memantine, and amantadine, were selected for a complete review. The second limitation is that the Y-BOCS scale was not used in all studies, though the OCD-VAS scale was used effectively with a similar measuring capacity for clinical effectiveness. There were limited results for amantadine, and most other NMDAR antagonist-focused studies were not found in the multi-database searches done for this review. Patient populations also represent a limitation of this review and could have resulted in biased results. Also, because the second literature search was conducted after the researcher lost access to the EBSCO/GALILEO database, this poses another limitation to this review.

Unlike the ketamine and memantine studies, which varied in medication status of the research subjects, the studies found with amantadine as the augmenting agent included already medicated patients. This evens the playing field in one aspect, as we can easily assess amantadine as an adjuvant only, but this does not show the power of amantadine alone. In future research, amantadine should be studied both alone and as an augment to assess its clinical power and compare it to other NMDAR antagonists.

## Conclusions

Ketamine is an effective glutamatergic agent for the treatment of mild OCD in non-refractory patients. There is still more research needed for ketamine as an anti-obsessive-compulsive agent, as it is known to provide a short-lasting response in most patients. Memantine and amantadine are efficient glutamatergic agents for the treatment of severe OCD, as adjuncts to SSRIs and without SSRIs. Considering the large fraction of the OCD population that is SSRI/SRI-refractory, these drugs may provide a new, severely needed treatment option for those who are unresponsive to SSRIs. There is limited research that studies amantadine as an augmentation agent, so there is a need to further this research.
