# Correction: Clinical Effectiveness of N-Methyl-D-Aspartate (NMDA) Receptor Antagonists in Adult Obsessive-Compulsive Disorder (OCD) Treatment: A Systematic Review

**DOI:** 10.7759/cureus.c328

**Published:** 2025-09-26

**Authors:** Asila A Ferguson, Aujala Irfan Khan, Baraa Abuzainah, Dipabali Chaudhuri, Kokab Irfan Khan, Roba Al Shouli, Akhil Allakky, Jaafar A Hamdan

**Affiliations:** 1 Psychiatry, California Institute of Behavioral Neurosciences & Psychology, Fairfield, USA; 2 Research, California Institute of Behavioral Neurosciences & Psychology, Fairfield, USA; 3 General Practice, California Institute of Behavioral Neurosciences & Psychology, Fairfield, USA; 4 Pediatric, California Institute of Behavioral Neurosciences & Psychology, Fairfield, USA; 5 Internal Medicine, California Institute of Behavioral Neurosciences & Psychology, Fairfield, USA; 6 Medicine, American University of Antigua, St. John, ATG

This article has been corrected to fix the following typos in the PRISMA diagram and article text:

- In the Abstract, "The database search yielded 4,221 articles" has been corrected to "The database search yielded 4,215 articles"
- In the 'Data Source and Strategy' section, first paragraph, "It yielded 4,221 results initially" has been corrected to "It yielded 4,215 results initially"
- In the PRISMA diagram, 'Records screened' and 'Records excluded' have been corrected from 135,627 and 135,608 to 135,623 and 135,603, respectively.
- In the Results section, first paragraph, "Nineteen studies consisting of..." has been corrected to "Eighteen studies consisting of..."

